# Smart Control and Energy Efficiency in Irrigation Systems Using LoRaWAN

**DOI:** 10.3390/s21217041

**Published:** 2021-10-24

**Authors:** Francisco Sánchez-Sutil, Antonio Cano-Ortega

**Affiliations:** Department of Electrical Engineering, University of Jaen, Campus Lagunillas s/n, Edificio A3, 23071 Jaén, Spain; fssutil@ujaen.es

**Keywords:** LoRaWAN, smart irrigation systems, smart energy

## Abstract

Irrigation installations in cities or agricultural operations use large amounts of water and electrical energy in their activity. Therefore, optimising these resources is essential nowadays. Wireless networks offer ideal support for such applications. The long-range wide-area network (LoRaWAN) used in this research offers a large coverage of up to 5 km, has low power consumption and does not need additional hardware such as repeaters or signal amplifiers. This research develops a control and monitoring system for irrigation systems. For this purpose, an irrigation algorithm is designed that uses rainfall probability data to regulate the irrigation of the installation. The algorithm is complemented by checking the sending and receiving of information in the LoRa network to reduce the loss of information packets. In addition, two temperature and humidity measurement devices for LoRaWAN (THMDLs) and an electrovalve control device for LoRaWAN (ECDLs) were developed. The hardware and software were also designed, and prototypes were built with the development of the electronic board. The wide coverage of the LoRaWAN allows the covering of small to large irrigation areas.

## 1. Introduction

In modern irrigated agricultural facilities, the competitiveness of the sector, combined with rising global temperatures, has necessitated the development of new and more sustainable agricultural techniques and crops to help reduce water consumption in these facilities, coupled with optimal water and energy management strategies. An efficient farming system is defined by the right amount of water at the right time, resulting in improved crop yields through efficient energy consumption. The use of innovative irrigation technologies is necessary to ensure an optimal amount of irrigation water. Optimisation of the irrigation system involves improving crop development conditions by planning the installation: optimal water and energy quantity and management. This requires variable monitoring and decision-making systems that allow us to optimise current irrigation installations.

The need for optimisation in agriculture began in the last century. In the beginning, design solutions with wired electronics were used but had numerous problems. Since then, the development and optimisation of irrigation systems have been linked to the rise and evolution of ICT (Information and Communication Technology). It is important to design sustainable models capable of supplying energy through renewable sources based on solar photovoltaic (PV) energy. Another fundamental part is given by the communication network, which is currently realised through wireless networks with low energy consumption, such as Low-Power Wide-Area Networks (LPWANs).

This article describes the design of an intelligent system to implement the irrigation control of a facility located on the university campus of the University of Jaén through wireless communication and low energy consumption powered by solar PV panels. This system consists of a wireless network with sensors and actuators that send the collected data, which are subsequently analysed in the cloud. This research focuses on optimising an irrigation system and reducing its energy consumption with an LPWAN supplied by a PV system.

## 2. Related Work

In the last decade, there has been a tendency to implement intelligent irrigation management systems based on wireless sensor networks, which have also been used in other areas such as industry, cities, housing, etc. The advantages of these wireless networks in the agricultural sector have been analysed by several authors, such as Goumopoulos et al. [1], who described the design of an intelligent system based on a wireless sensor and actuator network used for irrigation control in greenhouses. Doko Bandur et al. [2] analysed the energy consumption of the different components of the wireless sensor network, indicating the main energy consumers as well as how energy efficiency should be improved. The study of greenhouse crops with wireless technology used for sensor communication as well as the transmission rate was presented by Kochhar et al. [3]. Hamami et al. [4] reviewed the wireless sensor networks used in irrigation systems. This type of technology is ideal for system management and reducing water consumption.

Nowadays, the integration of devices with long-range wide-area networks (LoRaWAN) stores data in the cloud, where they are processed, analysed through Big Data and interact with other networks. These technologies enable the design of the Internet of Things (IoT) and cloud computing systems applicable to agriculture. Froiz-Míguez et al. [5] detailed an IoT system that develops a smart irrigation system covering large areas through a network (LPWAN) with soil temperature and humidity and air temperature sensors. Valente et al. [6] presented the development of a low-cost system and analysed energy consumption with a maximum of 400μA using a LoRaWAN network. Ameloot et al. [7] developed and analysed a wireless network with six wireless nodes to characterise the temperature and relative humidity of suburban areas using a long-range network (LoRa) at various locations in the city of Ghent (Belgium). It has also been used by Cano-Ortega et al. [8], who developed an optimal LoRa network using ABC algorithms to reduce Package Loss Rate (PLR) and dispatch time to determine the load profiles of a dwelling. Smart street lighting systems using an LPWAN control was realised by Sánchez-Sutil et al. [9,10]. Finally, Cruz et al. [11] monitored the filling level of urban waste containers in Lisbon (Portugal) using LPWAN technology. Ritesh-Kumar et al. [12] applied LoRaWANs to implement a greenhouse control system that enables energy and water savings through continuous monitoring of the installation.

The advances of ICTs in irrigation systems have been quite important, as can be seen. Nam et al. [13] discussed the use of ICTs in water management in agriculture and irrigation facilities. Goap et al. [14] presented an intelligent system that obtains soil moisture data through sensors together with current meteorological information to optimise the irrigation of an agricultural facility. To minimise water losses, Canales-Ide et al. [15] analysed a set of techniques and criteria aimed at optimal irrigation management that determines the water needs of plants and the optimal efficiency of the irrigation systems. By studying each plant from existing databases, Munir et al. [16] proposed an optimal irrigation system based on daily needs, considering the time of day, soil moisture and humidity. Migliaccio et al. [17] developed a smartphone application for scheduling urban lawn irrigation using evapotranspiration data from weather stations.

Among the most important advantages of using IoT systems in agriculture is automated irrigation, as measurements can be taken by sensors (humidity, temperature, irradiation, etc.) and actions (solenoid valves, pumps) through the different devices that make up this system. Methodologies have also been developed for the analysis and development of scientific networks based on the evaluation of the needs of different crops, soil attributes, climate, etc. Some authors have developed different IoT devices, as Fernández-Ahumada et al. [18] presented a low-cost device for automatic irrigation based on an ESP32-LoRa microcontroller and Internet connection through the Sigfox network. Fraga-Lamas et al. [19] proposed an IoT smart irrigation system specifically designed for remote urban areas. Chazarra-Zapata et al. [20] presented an IoT device that optimises battery power consumption, GPRS (General Packet Radio Service) with an Nb-IoT (Narrow-band Internet of Things) system for communication and sending information every two hours to reduce energy consumption. López-Morales et al. [21] proposed an IoT system that enables decision making on pumping efficiency in an irrigation community by easily integrating heterogeneous data sources, which improves the energy efficiency of pumping with higher economic, environmental and social returns in a sustainable way. Additionally, Glória et al. [22] developed a sustainable irrigation system that allows for improving natural resources, both water and energy, and reducing the economic cost through an IoT system with a network with batteries that has communication times of two hours. The monitoring of climatic parameters, soil moisture, vegetation health, plant diseases and crop yields while using IoT systems with wireless networks was developed by Khan et al. [23]. Additionally, Mohammed et al. [24] developed an IoT system for the control of date palms in arid regions using an underground irrigation system that remotely controlled climatic parameters and water volume in the soil. Tiglao et al. [25] presented a low-cost system that has a soil moisture sensor, a temperature sensor, a humidity sensor and a valve actuator within a mesh configuration that regulates drip irrigation. Finally, Sánchez Sutil et al. [26] performed the design of an intelligent system for measuring electrical variables to obtain load profiles in households.

Different control systems applied to irrigation have been developed. Al-Ali et al. [27] presented a microcontroller based on fuzzy logic algorithms for drip irrigation control, and Sudharshan et al. [28] studied a solenoid valve control system using fuzzy logic data from temperature, humidity and soil moisture sensors. Nawandar et al. [29] proposed a greenhouse, garden and farm control system and an automatic irrigation system capable of tracking the water needs of the crop, providing real-time and historical data of the farm. Liao et al. [30] performed the design of an automatic irrigation system with real-time soil moisture data to estimate the depth of water absorption. Finally, Eltohamy et al. [31] analysed how phosphorus released from the soil surface in paddy fields is influenced under different irrigation scenarios for different soil moistures.

The literature review found the following technological aspects.

- Wireless technologies are used in the works Wi-Fi [1,17,27,28,29,30], NRF [25], and RFID [13].
- Different works use LoRa technology [5,6,7,12,12,18,20,21].
- The algorithms used are optical algorithm [5], multi-objective function [18], and fuzzy logic [27,28].
- The following works use open-source platforms [5,13,29].

Based on the weaknesses and opportunities identified, the main contributions of this research are:

- Irrigation algorithm that connects to the Internet to obtain the probability of precipitation and does not irrigate if the probability of precipitation is greater than specified.
- Change of parameters in real time that allows the system to be much more dynamic and can be adjusted to the needs of the installation at any given moment.
- Routine checking of sending and receiving messages to minimise the number of packets of information lost in the LoRaWAN network.
- Development of low cost and open-source prototypes, which allow the system to be adapted to the particular needs of each installation.

The rest of the document is organised as follows: Section 3 provides an overview of the developed system, including the modular architecture of the system, the developed LPWAN network and the supported sensors and their interconnection with the platform. Section 4 details the technological results obtained. An evaluation of the developed system in terms of a prototype, the analysis of the performance of the LPWAN wireless network, the agronomic impact of the system and an evaluation of the energy consumption of the system are provided. Finally, Section 5 presents the conclusions drawn from this work.

## 3. Methodology and Design

### 3.1. Network Scheme

**Figure 1:**
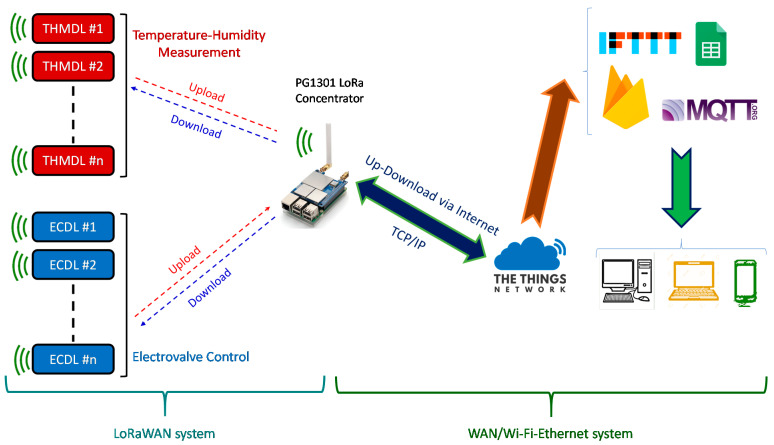
Proposed network scheme.

The proposed network scheme has two distinct parts. The first corresponds to the LoRaWAN and the second to the Wide-Area Network (WAN), which can be either wired using Ethernet protocol or wireless using Wi-Fi (Wireless Fidelity) protocol. The PG1301 concentrator manufactured by Dragino Technology Co., LTD., Shenzhen, China, is used as the link between the two networks. PG1301 is mounted on a Raspberry 3 or higher, which provides support for the WAN network. In addition, it can host up to 1000 LoRaWAN devices, which is sufficient for most applications. If a larger number is required, it is sufficient to install more concentrators to cover the required needs. Figure 1 shows the network scheme.

Within the LoRaWAN, communication is bidirectional between the Temperature and Humidity Measurement Device for LoRaWAN (THMDLs) and Electrovalve Control Device for LoRaWAN (ECDLs) with PG1301 since both data messages (upstream) and command messages (downstream) are needed. The information is concentrated on The Things Network (TTN) server [32]. This service is specially designed to work with LoRaWANs and supports upstream and downstream messages to LoRaWAN devices, such as the ones developed in this research (THMDL and ECDL). Currently, TTN has just implemented the new v3 version, which is much more powerful than the previous one. From TTN, it is possible to send and receive information to different IoT services through the available integrations. These include (i) AWS IoT [33], (ii) Akenza core [34], (iii) Datacake [35], (iv) deZem [36], (v) InfluxDB Cloud 2. 0 [37], (vi) Microsoft Azure [38], (vii) Qubitro [39], TagoIO [40], (ix) thethings.iO [41], (x) ThingsBoard [42], (xi) ThingSpeak [43], (xii) Ubidots [44] and (xiii) UIB [45].

In addition to the above integrations, it is possible to use other options such as (i) Message Queue Telemetry Transport (MQTT) [46], (ii) LoRa cloud [47], (iii) Node-RED [48] and If This, Then That (IFTTT) [49]. Finally, TTN has available the HTTP Webhooks integration that allows sending data to any server using POST and GET. From these, IoT services such as Google Sheets [50], Google Firebase [51], etc., can be accessed. As can be seen, there is a wide range of possibilities that allows the developer or user to find the service that best suits each situation at any given time.

### 3.2. Hardware Design

#### 3.2.1. Design Challenges and Objectives

In order to obtain fully functional devices that perfectly fulfil their assigned tasks, it is necessary to perfectly define the performance objectives to be met by the devices. These objectives will have a decisive influence on the choice of components and technologies to be implemented in THMDL and ECDL. They will also have an important bearing on the software that will run inside these devices. The hardware design objectives are listed below:

- Low power consumption: The devices are placed in the field (THMDL) or where the electrovalves are located (ECDL), and a mains power supply is not always available. It is necessary to use batteries and Solar Panels (SPs) to ensure the power supply of the equipment. In this sense, low power consumption is essential for batteries and SPs to be as small as possible.
- Small size: The devices must be installed in the smallest possible space. In the case of the THMDL, the goal is to be as imperceptible as possible in landscaped areas. For the ECDL, the goal is to be close to the electrovalve, but it is not always possible to have large spaces. This design objective is indivisibly linked to the previous one.
- Component integration, modular design and fault response: If one of the components has a problem and develops a malfunction, the device must be able to maintain the other features that have not been affected by the malfunction. The modular design is of vital importance in these scenarios since it allows components to be changed without the system ceasing to function. This results in highly fault-tolerant devices that provide a high degree of security against device malfunctions.
- Operational safety: The devices are designed to operate autonomously and continuously 24/7. It is, therefore, necessary for the design to be as robust as possible in order to minimise operating problems. This, together with the previous objective, gives the designed devices a high tolerance to failures.
- Low price: In addition to meeting all of the above objectives, the devices must have a final cost that is as low as possible. Thus, achieving designs that can be mass-produced and that are accessible to the majority of users is essential.

The aforementioned objectives entail overcoming a series of challenges and difficulties, the resolution of which will result in the development of fully functional devices. The following is the list of elements to be considered:

- Component selection: In achieving the design objectives, the selection of the components to be implemented in the devices is of particular importance. They have a decisive influence on the proper functioning of the devices and on achieving a final system that is fully functional and safe in its operation.
- Modular design: Combined with the design objectives of component integration, modular design, fault response and operational safety, the objective must provide robustness to the devices. Thus, devices cannot be taken out of service in the event of a malfunction. Rather, all features unaffected by the problem must continue to function correctly.
- Evaluation of alternatives leading to an optimal design: It is essential to evaluate the different implementation possibilities for each of the devices and the final system. The choice of the most correct, optimal and appropriate solution will lead to the fulfilment of the objectives set for the design.
- Printed Circuit Board (PCB) design: The design must be optimised to achieve a minimum size that allows the integration of all the selected components in each of the devices. In this case, two PCBs will be created, one for the THMDL and one for the ECDL.

#### 3.2.2. Components

An appropriate selection of the components to be implemented in the devices will have a decisive influence on the optimisation of the devices. The design objectives of low power consumption, small size, modularity and operational safety must be addressed in the approach to component selection.

##### Microcontroller

In order for a device to function properly and perform all assigned tasks, it is necessary to have a core. This core is the microcontroller that must drive the interaction between components within the device. Thus, the microcontroller must have different elements such as the microprocessor, memory, ports for communications with other components, digital and analogue inputs and outputs, etc.

Once the design objectives have been studied, it can be concluded that the Arduino family of microcontrollers is an ideal platform for use in the construction of the devices created in this research. The Arduino platform is endorsed by its use in a multitude of projects with industrial and domestic applications.

Table 1 shows some of the most essential features of the various microcontrollers in the Arduino family. This table is the basis for choosing the microprocessor applied to the devices developed in this research.

The Arduinos shown have sufficient memory capacity for program code and data. Therefore, the decision to use one or the other microcontroller depends on other design objectives, which are mainly power consumption and size. In this sense, the microcontroller that best meets the requirements defined in the design is the Arduino Nano (AN), whose specifications can be found in [52].

##### LoRa Wireless System

Regarding the LoRa communication system, it should be noted that two components are required: (i) the end device (to be installed in the THMDL and ECDL) and (ii) the gateway responsible for communication with the cloud.

LoRa communication chips are diverse, including (i) Semtech (Semtech Corporation, Camarillo, CA, USA) SX1308 [56], SX1301 [57], SX1276 [58], SX1278 [58] and SX1257 [59]; (ii) HOPERF chip RFM95/96/97/98 [60] (HOPERF, Shenzhen, China); and (iii) Murata CMWX1ZZABZ (Murata Manufacturing, Nagaokakyo, Japan) [61].

Commercially available LoRaWAN-compatible models are built from these chips. Five models were analysed. From this analysis, the model to be implemented in the THMDL and ECDL was chosen. The models analysed were the following: (i) Arduino MKRWAN 1310 (Arduino AG, Ivrea, Italy) [62]; (ii) Monteino (LowPowerLab, Canton-Michigan, USA) [63]; (iii) Libelium (Libelium, Zaragoza, Spain) [64]; (iv) Lopy4 (Pycom, Bucharest, Romania) [65]; and (v) Dragino LoRa Bee (DLB) (Dragino Technology Co., LTD., Shenzhen, China) [66]. These models use the following chips: (i) Murata CMWX1ZZABZ for the Arduino MKR WAN 1310; (ii) HOPERF chip RFM95/96/97/98 for the Monteino; (iii) SX1276 and SX1278 for the Lopy4 and DLB, respectively; and (iv) Semtech SX1272 for the Libelium. The characteristics of the models analysed are similar. Therefore, the DLB was chosen in this research as the component to be installed in the THMDL and ECDL due to its reduced price. Table 2 illustrates the characteristics of the components analysed.

Once the LoRa component was selected, it was necessary to choose the gateway. This component is responsible for handling the upstream and downstream messages sent back and forth between the THMDL and ECDL to TTN. Although there are different options on the market, we chose to use the Dragino family to ensure better compatibility with the chosen LoRa component. Table 3 shows the four gateways tested, from which the LoRa PG1301 [67] concentrator was chosen. PG1301 is capable of handling up to 1000 devices with 10 channels of communication, which is more than enough for most systems. If one needs to control more than 1000 LoRa devices, additional gateways can be added. PG1301 was mounted on a Raspberry Pi computer that provides support for Internet access, either via Ethernet cable or Wi-Fi.

##### Electrical Variables Meter

For the measurement of DC variables, there are fewer options with sufficient quality. There are three possibilities: (i) FZ0430 [71]; (ii) ACS712 [72]; and (iii) INA219 [73]. FZ0430 is capable of measuring only voltage up to 25 V in direct current. ACS712 measures currents in ranges of 5, 20 or 30 A, depending on the version. To obtain the power consumption, it is necessary to include a unit of these sensors and then perform the necessary calculations. A complementary option is to use the INA219 m, which is capable of measuring voltage and voltage in the same component. It also provides a direct reading of the power consumed. In this research, INA219 has been chosen as it involves making all voltage, current and power measurements on the same component. The characteristics of the components analysed are shown in Table 4.

##### SHT30 Temperature and Humidity Sensor

After searching for temperature and humidity sensors that could work with the Arduino platform, five families of sensors were found. These families are the following: (i) SHT1x [74]; (ii) SHT2x [75]; (iii) SHT3x [76]; (iv) DHT11 [77]; and (v) DHT22 [78]. Measurement ranges, accuracy, power consumption, supply voltage and communications paths are diverse. Table 5 shows the comparison of the analysed sensor models.

It can be seen that the family offering the best performance is the SHT3x. Accuracy, power consumption and measurement ranges are outstanding. Moreover, the supply voltage and the I2C (Inter-Integrated Circuit) bus are ideal for use in conjunction with AN. Finally, within the SHT3x family, the SHT30 sensor was chosen for implementation in the THMDL.

##### Charge Regulator

SeeedStudio controllers offer a wide range of use in the charge control of batteries with SPs. Of the three models analysed, the Lipo Rider Pro (LiPo) [79] model was chosen for implementation in the devices. This model offers ideal characteristics for the 3.7 V battery and the 4.8 V SP used. Moreover, it is also perfectly suited to the supply voltage of the AN board. Table 6 shows the characteristics of the models tested.

##### Solar Panel

Solar energy is clean, renewable and simple to use. In this sense, it is of great interest to be used as a source of energy for equipment working outdoors, such as those used in this research. The chosen SP has a high transformation efficiency of around 17%. It is made of monocrystalline material and coated with a thin layer of resin on the surface that protects it from atmospheric agents and makes it ideal for outdoor use. The dimensions of the SP are 138 × 160 mm. The nominal output voltage is 5.5 V, with an output of 540 mA, depending on the luminous intensity received. The open-circuit voltage is 8.4 V, and the maximum load voltage is 6.4. The main characteristics can be found in [82].

##### Battery

For use in THMDL and ECDL, a 3.7 V, 7800 mAh, 28.86 Wh lithium-ion battery was chosen. This is more than enough to power the designed devices. It should be noted that the THMDL has an average consumption of 166.5 Ah, which ensures 31 h of operation with a fully charged battery. In the case of the ECDL, the average consumption is 31 Ah, with a battery life of 174.5 h if the battery is fully charged.

The battery pack has dimensions of 68 × 55 × 19 mm and consists of three individual batteries, with an operating temperature of between −20 °C and +60 °C. The characteristics of the battery are available for consultation in [83].

#### 3.2.3. Hardware Implementation for the THMDL

In the design of the THMDL, two AN microcontroller was used. This is because the communications paths system chosen (LoRaWAN) and the chip that gives access to the DLB are not compatible with the I2C communications paths bus used to read the INA219 and SHT30 sensors. To perform the measurements, AN1 sends the reading request via the serial port, and AN2 performs the measurement of electrical variables, temperature and humidity and returns them via the serial port. Therefore, AN1 takes care of the communication with the LoRaWAN network and controls the measurement request, and AN2 takes care of the necessary measurements.

Due to the fact that the devices developed in this research work autonomously with no possible wired connection to the electrical and Ethernet networks, it is necessary to implement power supply systems and access to communications paths that do not depend on wired networks. For the electrical network, two solutions have been considered: (i) a battery and (ii) a battery and SP controlled by a regulator. This results in two different versions of the THMDL. The aim is to monitor the power consumption of the joint system and the contribution of the battery and the SP in order to control the system and replace or charge the battery to keep the system running.

For the implementation of the wireless system, there are several applicable technologies. These include the following: (i) Bluetooth; (ii) SigFox; (iii) ZigBee; (iv) Nb-IoT; and (v) Wi-Fi. The coverage offered by each of these is variable, in many cases not exceeding tens of metres, as well as requiring repeaters to extend their coverage. SigFox is owned by a company, which is why all services must be contracted with it. NB-IoT requires a data contract and a SIM card to send and receive information.

**Figure 2:**
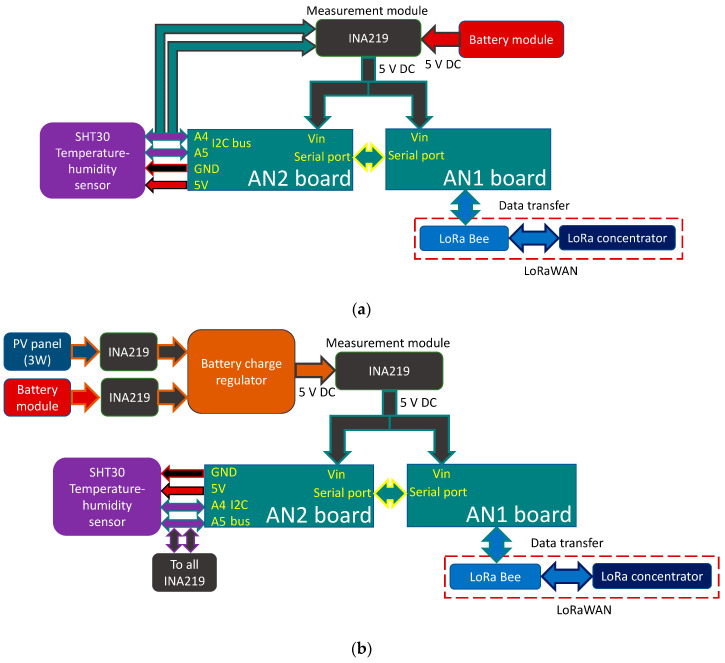
Block diagram of the THMDL: (**a**) battery power supply and (**b**) battery and solar panel power supply.

This research uses LoRaWAN because communications paths can be achieved up to 10 km, with an average of 5 km, which are sufficient distances for application in most landscaped areas in cities. If larger extensions are required, it is only necessary to install more gateways to ensure the necessary system coverage. Figure 2 shows the block diagrams of the two versions of the THMDL. These diagrams express the relationships that are established between the different components of the devices and how they share information and electrical characteristics.

**Figure 3:**
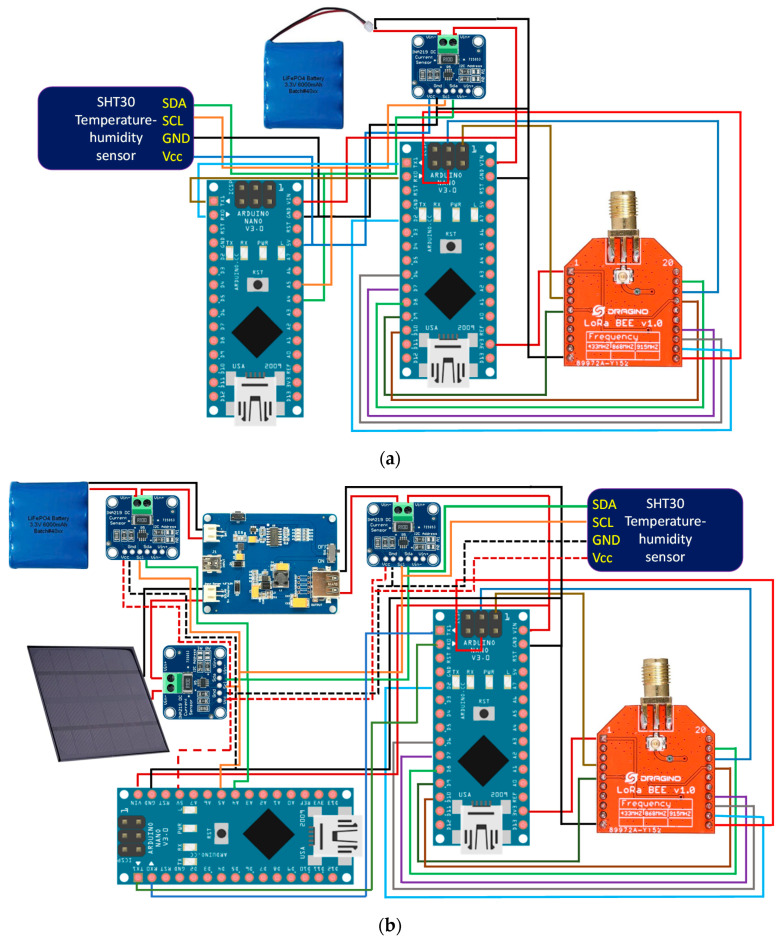
Wiring diagram of the THMDL: (**a**) battery power supply and (**b**) battery and solar panel power supply.

To complement the block diagrams, the wired connections made between the different components are included. This allows any researcher to be able to clone the devices presented in this research. The power supply, serial port communication, I2C bus communication and LoRaWAN network connections can be seen in the block diagrams. Figure 3 shows the wiring diagram for the THMDL in both versions.

**Figure 4:**
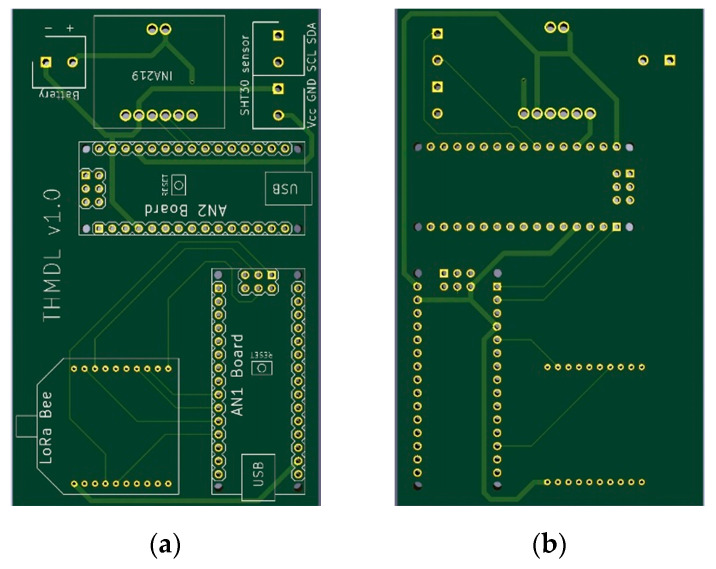
PCB of the THMDL with battery power supply: (**a**) front side and (**b**) back side.

**Figure 5:**
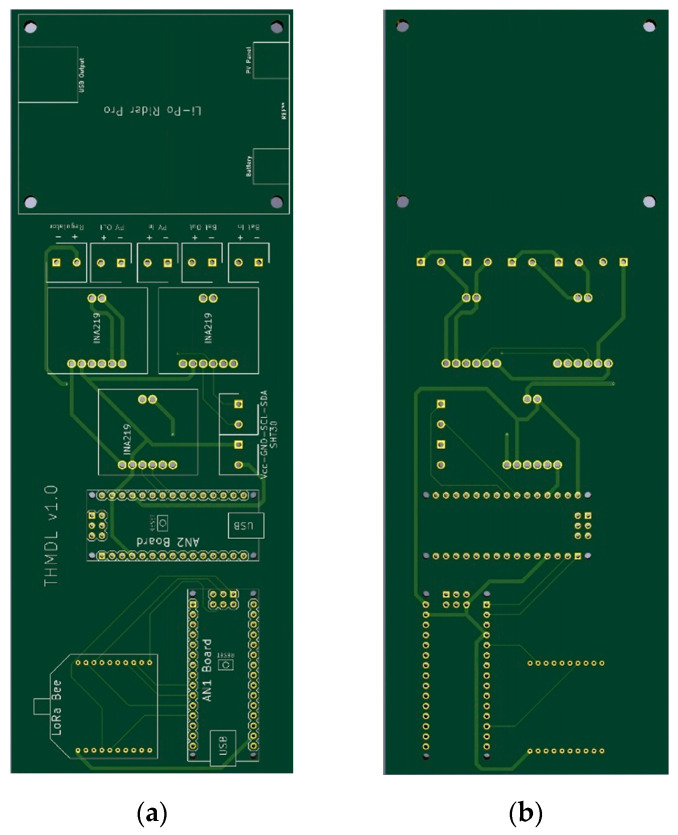
PCB of the THMDL with battery and solar panel power supply: (**a**) front side and (**b**) back side.

**Figure 6:**
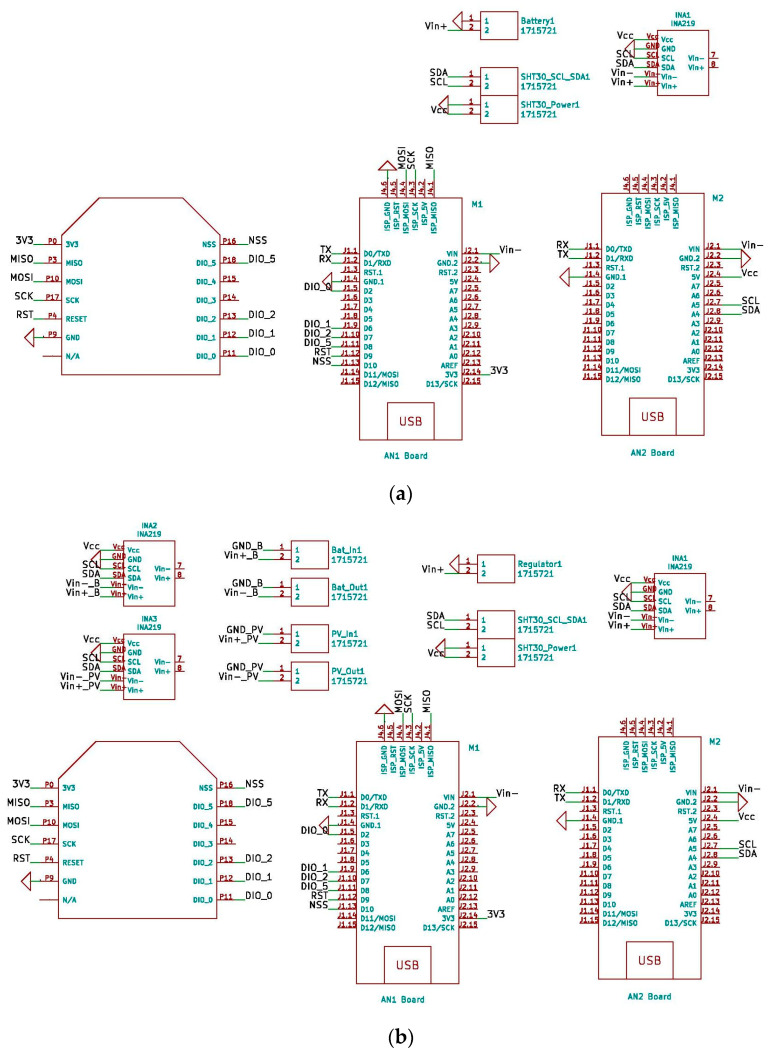
Schematic of the THMDL: (**a**) battery power supply and (**b**) battery and solar panel power supply.

The THMDLs PCB board was also designed in its two versions: battery power supply and battery power supply plus SP. The board integrates all the components used, avoids wiring as much as possible and gives solidity to the whole. The dimensions are 95 × 58 mm for the battery-only version and 191 × 71 mm for the version with battery and SP. Figure 4 and Figure 5 show the design of the PCB boards. Figure 6 shows the electronic schematic of the PCB board for THMDL.

It is important to perform the economic valuation of the THMDL in its two versions to check whether the reduced-price target is met. In this regard, Table 7 and Table 8 show the price of each product and the price of the final set for the two versions of the THMDL. It should be noted that the price of the products is obtained from the official shops of the manufacturers. On the other hand, the fact that the components are licence free means that there are compatible components on the market that can further reduce the price of the set.

#### 3.2.4. Hardware Implementation for the ECDL

Similar to the THMDL, the ECDL has two ANs due to the incompatibility of the I2C bus with the LoRaWAN system. In this case, a relay is available to operate the electrovalve. This relay is connected to digital output 3 of AN1. The supply voltage of the relay is therefore 5 V, and it can withstand currents of up to 10 A.

AN2 takes care of the electrical measurements made by the INA219. In the SP versions of the THMDL and ECDL, 3 INA219 m are required. It is, therefore, necessary to assign an address on the I2C bus in order to be able to read the contents of each one. The INA219 output meter of the charge controller is assigned the default address 0 × 40. The battery measurement INA219 was assigned the address 0 × 41. For this reason, it is necessary to make a bridge between the A0 contacts of INA219. Finally, the INA219 m of the SP was assigned the address 0 × 44, bridging, in this case, the two A1 contacts. This allows access to the individual measurements without any interference between the measuring device addresses.

**Figure 7:**
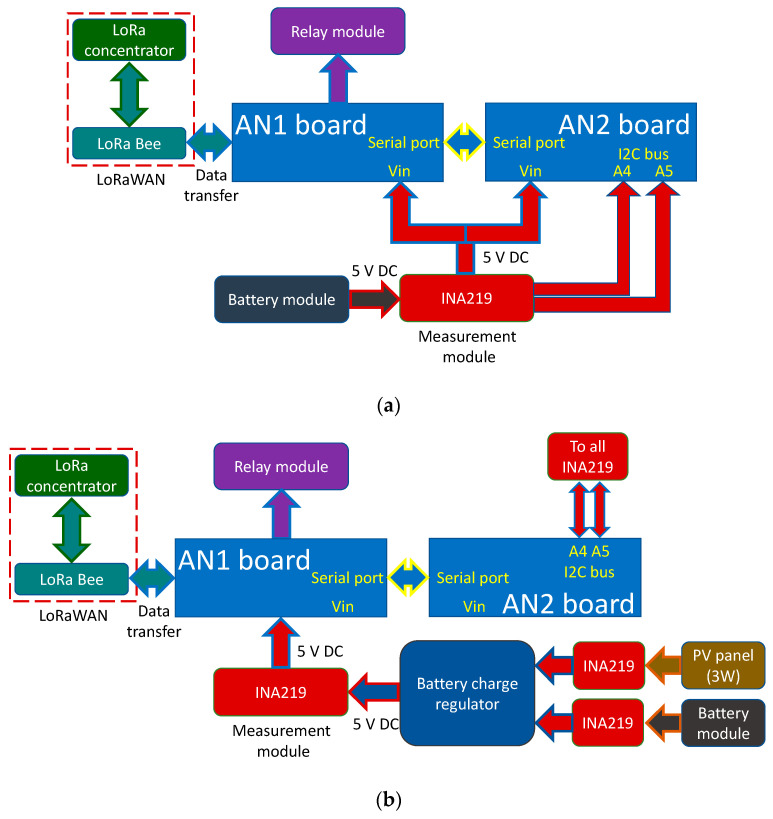
Block diagram of the ECDL: (**a**) battery power supply and (**b**) battery and solar panel power supply.

The system for access to the LoRaWAN network via the DLB is the same as explained for the THMDL. The difference is the messages exchanged with the network because the ECDL performs different functions. To understand the functional relationships between the components used in the two versions of the ECDL, see Figure 7.

**Figure 8:**
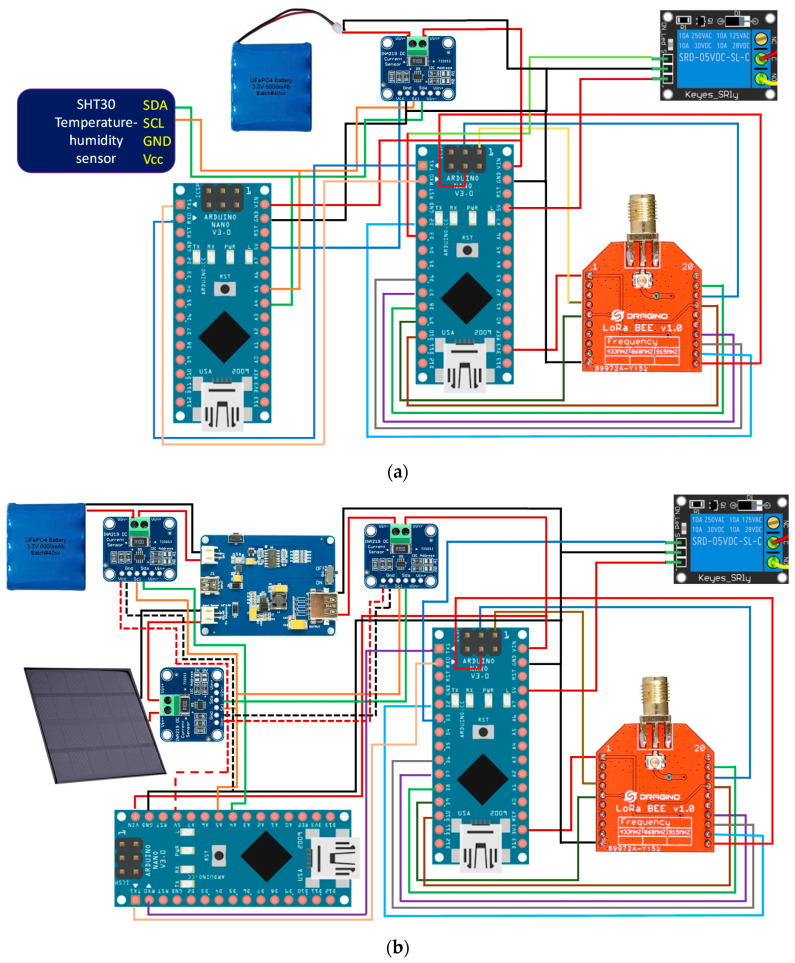
Wiring diagram of the ECDL: (**a**) battery power supply and (**b**) battery and solar panel power supply.

For the ECDL, it is also necessary to include the wiring diagrams that show the electrical connections to be made in order to build this device. The wiring diagrams are complemented with the block diagrams for a complete definition of the ECDL. With all the information provided, the ECDL is reproducible for any interested researcher. Figure 8 shows the wiring diagrams for the ECDL in its two versions.

**Figure 9:**
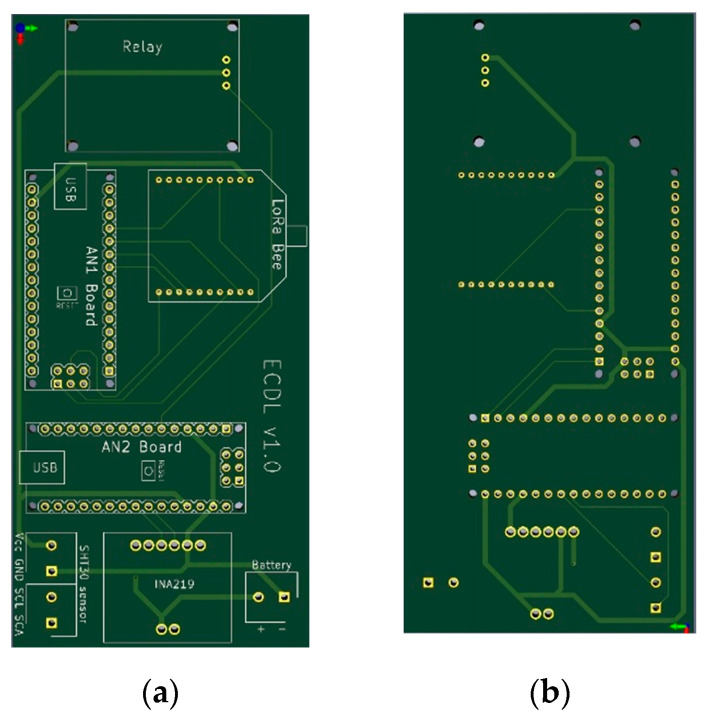
PCB of the ECDL with battery power supply: (**a**) front side and (**b**) back side.

**Figure 10:**
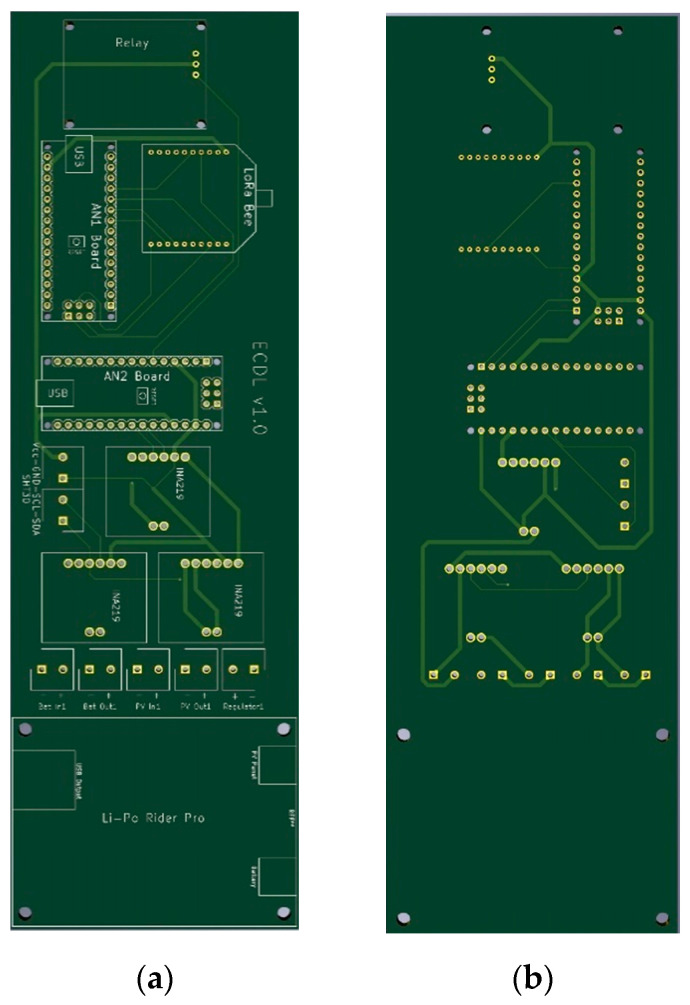
PCB of the ECDL with battery power supply: (**a**) front side and (**b**) back side.

**Figure 11:**
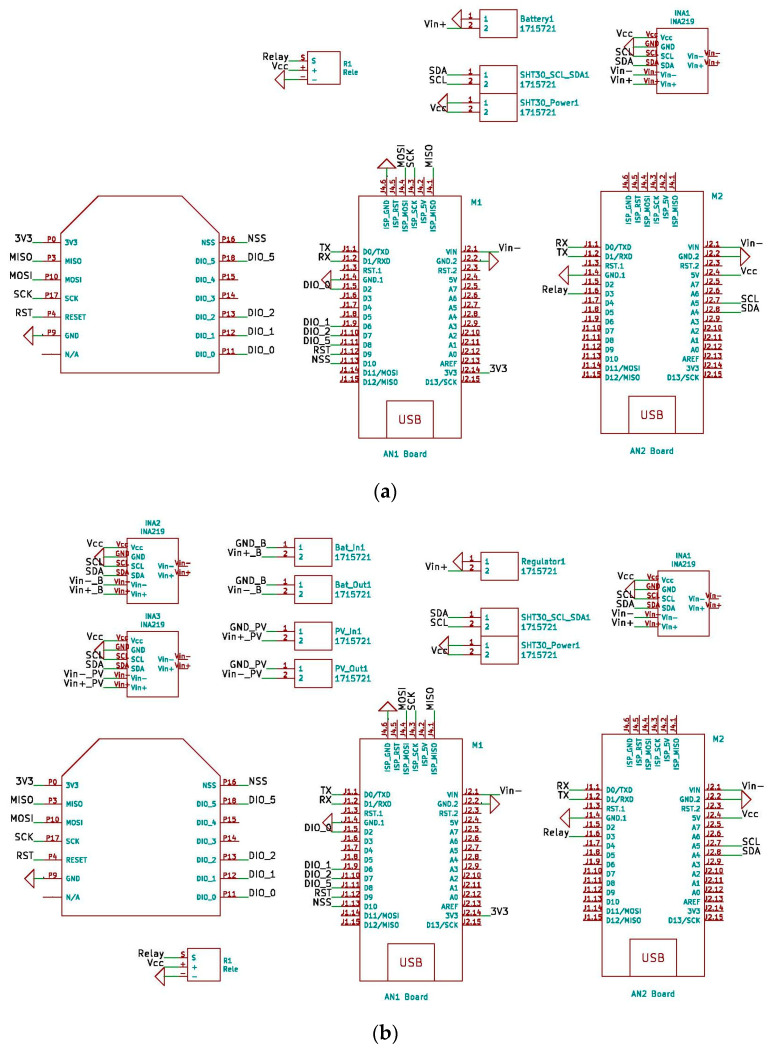
Schematic of the ECDL: (**a**) battery power supply and (**b**) battery and solar panel power supply.

Finally, Figure 9 and Figure 10 show the electronic board developed for the ECDL in its two versions. In this case, the dimensions of the boards are 125 × 59 and 220 × 70 for the ECDL versions with and without an SP. The electronic schematic of the PCB board for THMDL is shown in Figure 11.

The economic valuation for the ECDL versions has also been done to verify that the reduced-price target has been met. Table 9 and Table 10 give the approximate cost of the two ECDL versions.

### 3.3. Software Design

The system designed in this research is intended to operate continuously in a 24/7 mode. This allows the automated system to be permanently under control. In addition, the devices have to perform all their functionalities again when there is any problem, e.g., battery change.

Several functionalities have been implemented in the system: (i) battery charge level control; (ii) watering routine based on the weather forecast and humidity level; (iii) complete electrical (v, I, p) and environmental (temperature, humidity) measurements; and (iv) parameter change.

#### 3.3.1. THMDL Software

The THMDL program is structured in two main sections: (i) initialisation and (ii) command control. The initialisation tasks must prepare the components used and the communication ports to start the continuous process.

The command control routine must continuously scan the network for messages sent from the system. These messages are of two types: (i) measurement message and (ii) irrigation message. When the measurement message is received, the measurement routine that measures the electrical and environmental parameters is executed. Subsequently, the battery check routine is executed to check the state of charge of the battery. If the message sent is for irrigation, the need to irrigate is checked, and the necessary order is sent to the system.

**Figure 12:**
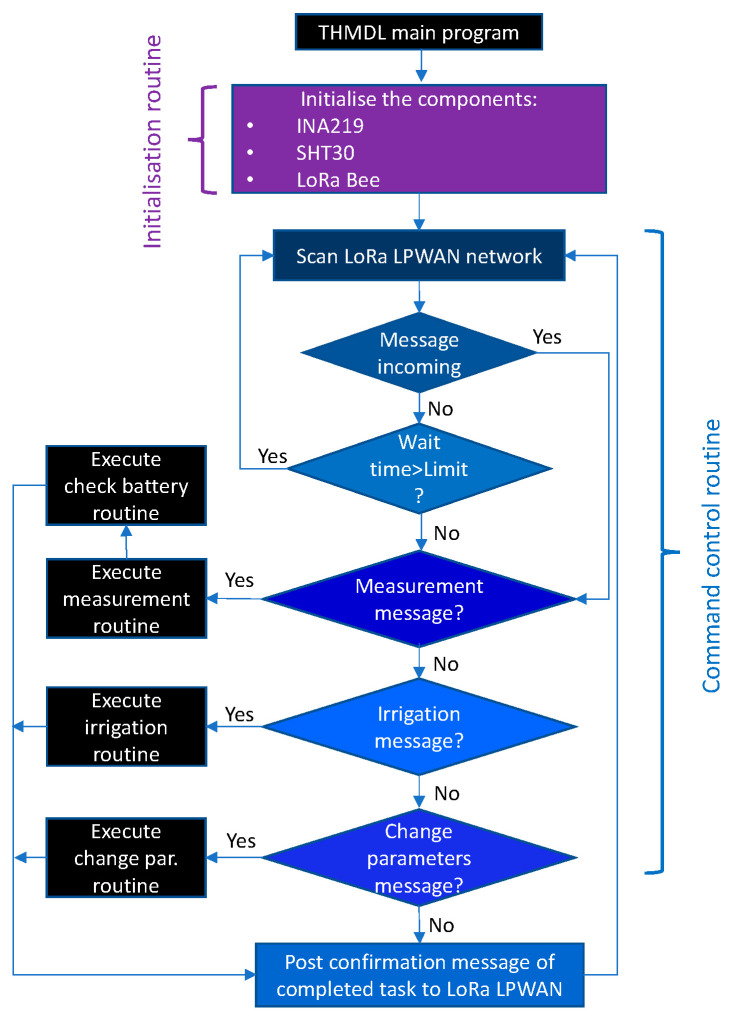
Flow chart of the main program for the THMDL.

Once the task required by the system has been performed, a task completed confirmation message is sent so that the system is notified of the completion of the task. Once the confirmation message is sent, the system returns to the initial step of scanning the LoRaWAN network for new messages. The above process for the command control routine is performed continuously as long as the THMDL is connected. Figure 12 shows the flowchart for the main THMDL program.

**Figure 13:**
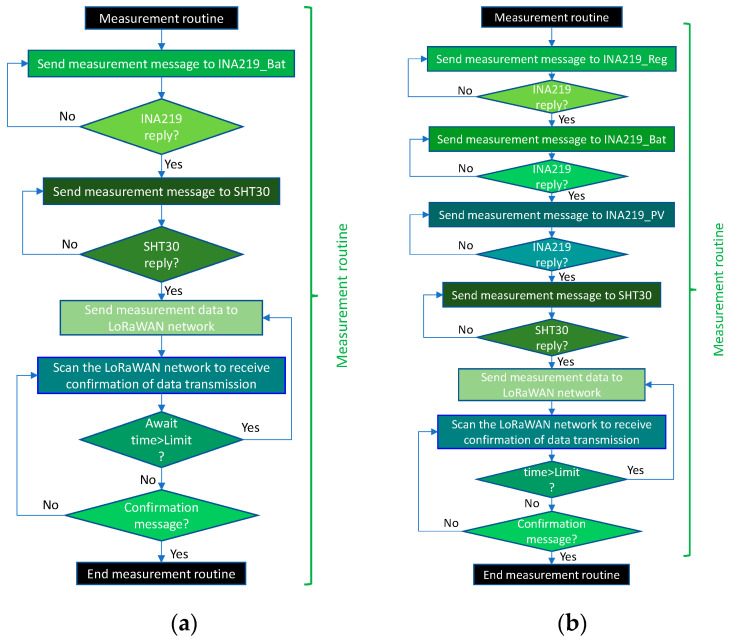
Flow chart of the measurement routines for the THMDL: (**a**) battery power supply and (**b**) battery and solar panel power supply.

Figure 13 shows the flowcharts for the measurement routines in the two versions of the THMDL. The routine is divided into two parts: (i) measurement of electrical variables and (ii) measurement of environmental variables. First, the measurement phase of the electrical part is performed. In this phase, the corresponding INA219 m is called, which returns the variables *v*, *i* and *p*. In the case of the battery-only version, only one measurement is taken. The version with an SP performs three measurements in this order: (i) regulator, (ii) battery and (iii) SP.

Once the measurement of electrical variables has been completed, the measurement of environmental variables is performed. To do this, SHT30 is called and returns the temperature and humidity data recorded at that moment. Finally, all the measured data are sent to the LoRaWAN network. In this sense, a message security system has been implemented in order to minimise the loss of information in the system.

**Figure 14:**
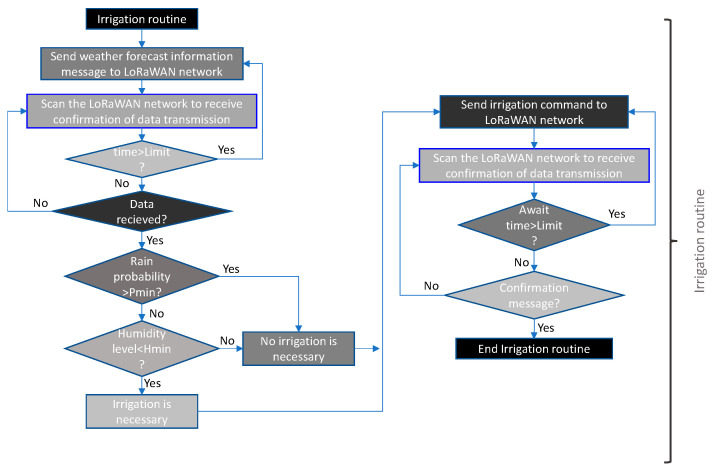
Flow chart of the irrigation routine for THMDL.

Figure 14 shows the flow chart of the irrigation routine. It is important to note that this routine has been implemented based on weather forecasts so that water expenditure is minimised. When the routine is started, a request for rainfall forecast data is sent. The forecast can be extended over the necessary time horizon to be estimated in each application. Once the probability of precipitation is received, it is compared with the minimum probability assigned. If the probability received is higher, the message that it is not necessary to irrigate is sent. On the other hand, if the probability of precipitation is lower than the minimum, it is passed to the part of comparison with the measured humidity level.

If the humidity level is lower than the minimum level set for watering, the watering message is sent. Otherwise, the message that watering is not necessary is sent. The levels of precipitation probability and minimum humidity can be changed by the user at any time, making the system much more efficient and dynamic.

**Figure 15:**
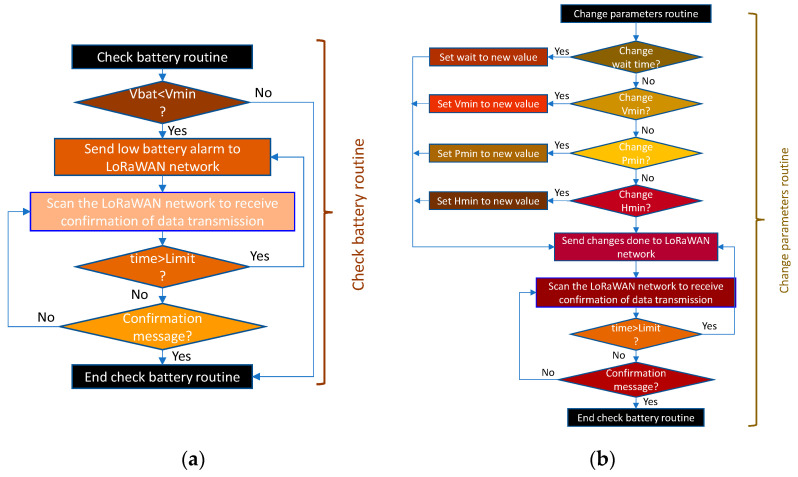
Flow chart of the (**a**) check battery routine and (**b**) change parameters routine for the THMDL.

The battery check routine is shown in Figure 15a. Here, it can be seen that the check is performed in relation to the battery voltage. If the above voltage falls below the defined minimum, a low battery message is sent to the system for action by the maintenance staff.

To make the system more dynamic, it is necessary to be able to change the action limits at any time. This allows it to adapt to new situations or approaches in system policies. It also allows sectorisation of the system, making it possible to have different parameters in each zone reflecting the particular characteristics. For this purpose, Figure 15b shows the THMDL parameter change routine. Parameters can be changed together, either individually or in groups, as the routine is prepared for this.

#### 3.3.2. ECDL Software

**Figure 16:**
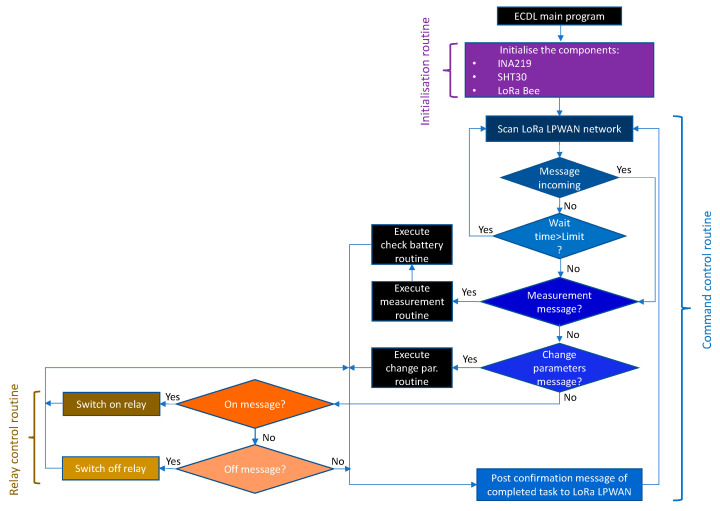
Flow chart of the main program for the ECDL.

The design of the main ECDL programme follows a similar philosophy to that of the THMDL. It starts with the initialisation phase of all components. Subsequently, in continuous mode, it executes the following tasks: (i) scanning the LoRaWAN network for new messages; (ii) if a measurement message arrives, it performs the measurement, checks the battery state of charge and sends data; (iii) if a parameter change message arrives, it executes the parameter change routine and sends confirmation; (iv) if the message is an electrovalve on-or-off message, it activates or deactivates the relay. Figure 16 shows the flowchart for the main ECDL program.

**Figure 17:**
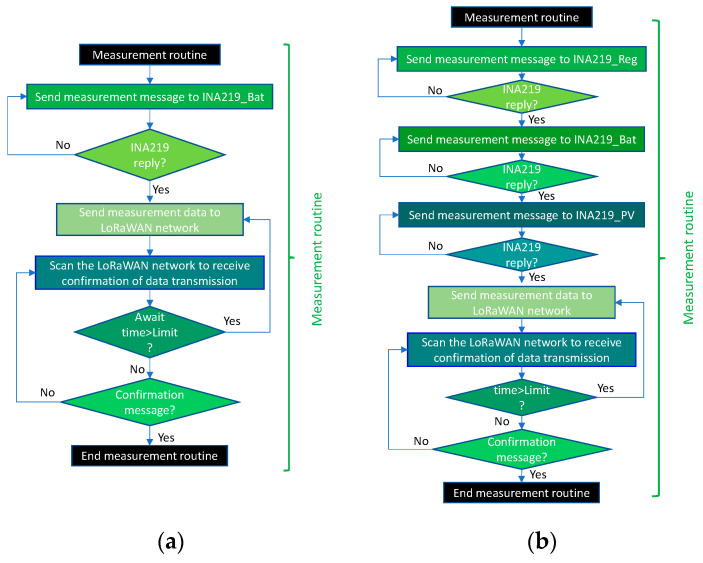
Flow chart of the measurement routines for the ECDL: (**a**) battery power supply and (**b**) battery and solar panel power supply.

Figure 17 shows the flowcharts of the measurement routines for the two versions of the ECDL. The routines work in the same way as those shown above for the THMDL. In this case, the temperature and humidity measurement part is omitted, as this device does not need to take these measurements. It is also enabled to send messages until receiving confirmation of the arrival of the data.

**Figure 18:**
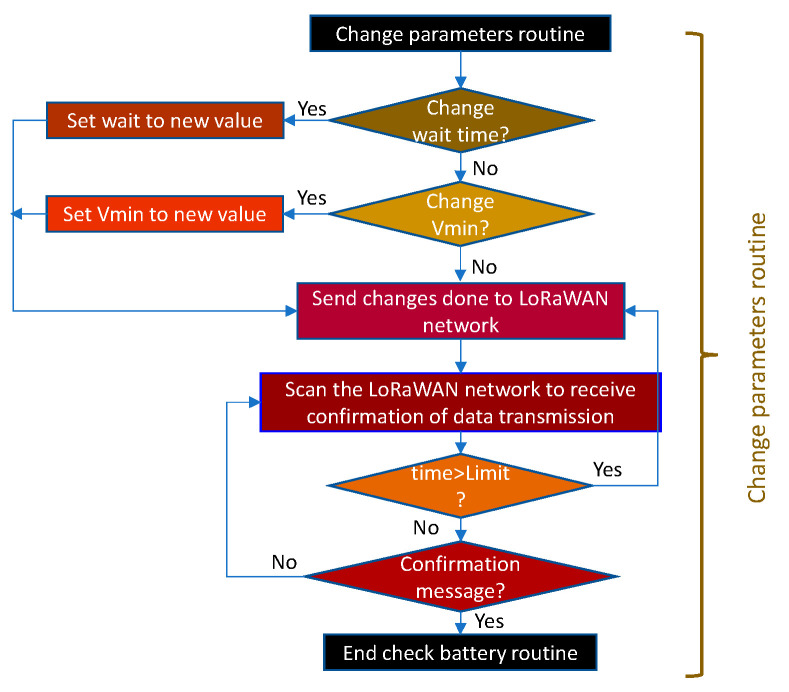
Flow chart of the change parameters routine for the ECDL.

As with other routines, the data modification routine is based on the one described for the THMDL. In this case, only two variables are needed: (i) timeout for receiving and sending messages and (ii) minimum voltage for sending low battery warnings. Figure 18 shows the parameter change routine for the ECDL.

## 4. Results and Discussion

This section shows the tests conducted to check the devices created in this research. Data were collected in Jaén, Andalusia, Spain, during different times of the year in order to validate the design and implementation.

### 4.1. Case Study

**Figure 19:**
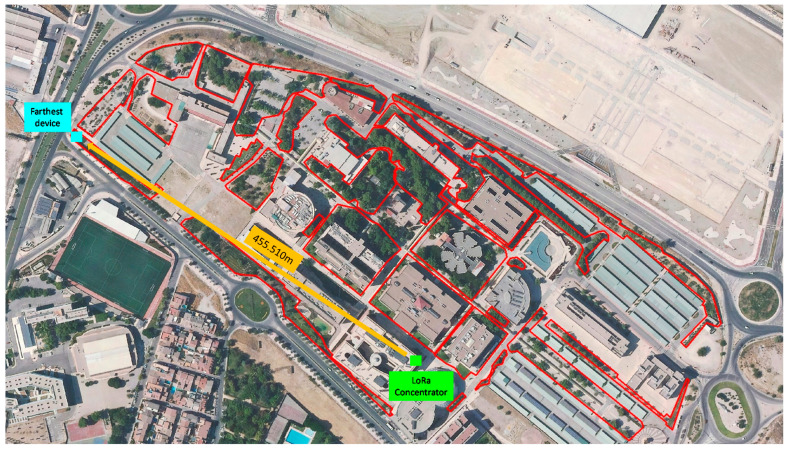
Distribution of irrigation zones on the campus.

Figure 19 shows the distribution of irrigation zones on the campus. The number of zones is 22, in which a THMDL device for temperature and humidity measurement has been installed in each zone. An ECDL actuator has also been installed in each zone to control the irrigation electrovalve. This allows the areas to be separated and only irrigate those that really need water. This avoids unnecessary water wastage in areas with sufficient humidity.

For applications in cities, the necessary zones will be distributed according to the characteristics of the area to be irrigated automatically. In each zone, a THMDL device must be installed to monitor the zone. The THMDL will communicate with the LoRaWAN system, sending all the recorded data and irrigation orders required. As for the ECDL devices, these will be installed in each of the electrovalves that irrigate the automated zones. It may happen that several zones monitored with the THMDL are irrigated with the same electrovalve. Therefore, the number of THDML devices does not always coincide with the number of ECDL devices. Aerial photographs, photographs taken with drones, maps of the area, etc., can be used to perform the study. These tools allow a detailed study of the area to be done and the best possible system to be implemented.

### 4.2. LoRaWAN Configuration

In this research, we chose to send messages every minute, which is sufficient for an installation of this type. The initial configuration chosen in this case is BW125 (Band Width 125 kHz), SF7 (Spreading Factor) and CR4/5 (Code Rate). The length of the payload is 16 bytes plus 13 bytes for the header. The 16 bytes of the payload are distributed in 2 bytes for each variable, 2 bytes for temperature, 2 bytes for humidity, 2 bytes for battery voltage, 2 bytes for battery current, 2 bytes for PV module voltage, 2 bytes for PV module current, 2 bytes for bus voltage and 2 bytes for bus current. The header layout is 1 byte for MAC header (MHDR), 4 bytes for LoRaWAN device address, 1 byte for FCtrl (control bit), 2 bytes for Fcont (count bits), 4 bytes for the message integrity code (MIC) and 1 byte for Fport (port number).

Due to the 60 s send time and the chosen payload length, it is only possible to use SFs between 7 and 10, with a 1% duty cycle duration of 41.2 s for SF10 and less for the other SFs. SF7 is chosen as it has the shortest duty cycle duration. The location of the installation is in Europe, so only BW125 and BW250 are possible. The speed of BW250 is higher, but the transmission distance is shorter. To ensure less PLR with the existing distances, a BW125 has been chosen.

Table 11 shows the calculation of the on-air times for the header and payload used. Here, you can see the minimum time for sending messages according to the 1% duty cycle. With the chosen configuration, it is 6.7 s. Table 11 shows all possible combinations for the EU868 zone in Europe. In other regions of the world with different frequency plans, other results can be easily obtained.

### 4.3. Measurement of Soil Temperature and Humidity

This section shows the temperature and humidity measurements for all days of the four seasons taken in one of the zones in the year 2020. It can be seen that temperature increases and humidity decreases in the seasons of the year. The season with the highest average temperature corresponds to summer with an average of 29.31 °C, the lowest average temperature of 12.14 °C and the annual average of 19.03 °C.

**Figure 20:**
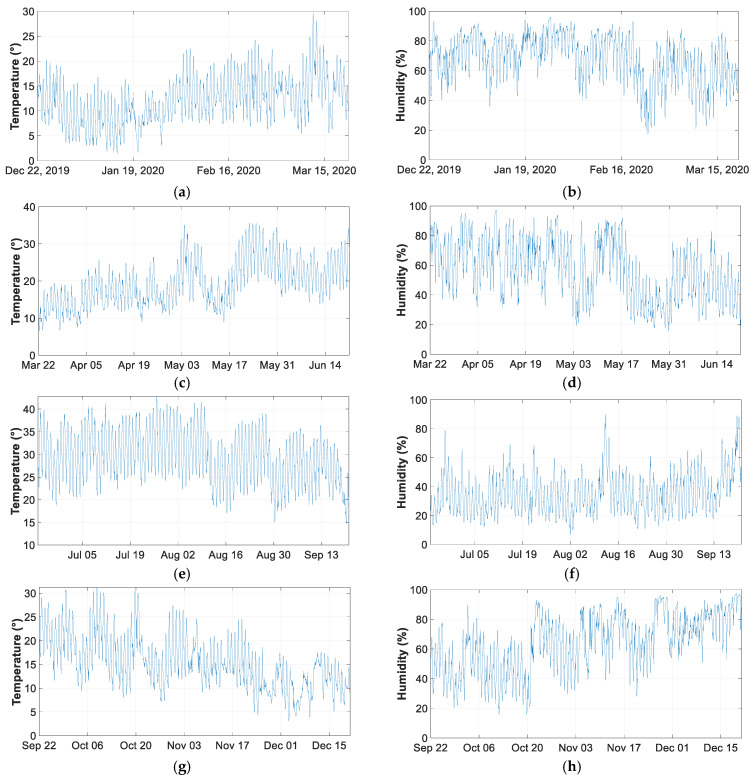
Temperature and humidity graphs for the year 2020: (**a**) temperature in winter; (**b**) humidity in winter; (**c**) temperature in spring; (**d**) humidity in spring; (**c**) temperature in summer; (**f**) humidity in summer; (**g**) temperature in autumn; and (**h**) humidity in autumn.

The highest average humidity occurs in winter with a value of 67.81%, the lowest average in summer with 35.40% and the annual average is 56.09%. Figure 20 shows the data obtained during the meteorological stations of the year 2020.

The location of the campus is defined by its UTM coordinates referenced to zone 30: X = 431,582 and Y = 4,182,595. The geographical location of Jaen has a continental Mediterranean climate. As it is located near the Guadalquivir river valley, this has a decisive influence on the climatic conditions. The temperature variation that can occur is around 20 °C.

### 4.4. Battery Charge

It is possible to charge the battery in two different ways: (i) via the USB port and (ii) via the SP. The first option is possible via the mini USB port of the LiPo.

**Figure 21:**
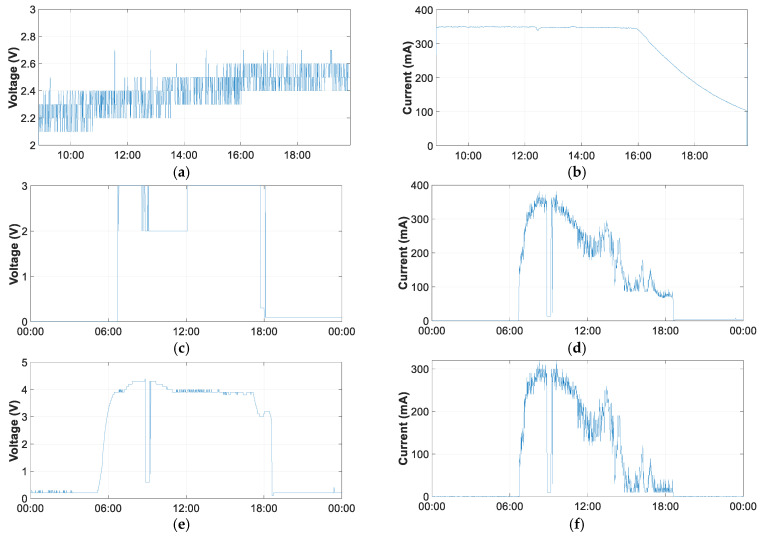
Electrical variables measurement in battery charge: (**a**) battery voltage in USB charge; (**b**) battery current in USB charge; (c) battery voltage in PV charge; (**d**) battery current in PV charge (**e**) PV voltage in PV charge; and (**f**) PV current in PV charge.

This port can be connected to various devices, such as a computer USB port, a mobile phone charger, or any other type of charger that provides 5 V and 500 mA DC. Figure 21a,b shows the voltage and current of a complete charging process through the USB port, in this case, of a laptop. From the voltage curve, it can be seen that the voltage increases as the accumulated battery charge increases. The voltage evolves from 2.2 V to 2.6 V at the end of the charging process. The charging current averages 350 mA until the seventh hour of charging. Thereafter, the current decreases to 100 mA at the end of the charging process, which drops abruptly to zero when the battery is fully charged. The complete charging process takes 10 h and 50 min.

Figure 21c–f shows the battery charging process using the SP. It can be seen that although the SP is live, LiPo only switches on the battery charging when the SP voltage is close to 4V. The battery charge remains constant at 3 V, with some periods of 2 V. The charging current depends on the radiation that the SP is receiving. A voltage drop is also observable around 10 am due to passing clouds.

As can be seen, charging the battery with a USB is much faster and more recommendable when the battery is discharged. With a full day of charging with the SP, the battery was not fully charged. This affirms that the function assigned to the SP is to extend the duration of the battery charge by providing charging during sunshine hours. Thus, the SP replenishes the energy consumed during the night and makes the equipment autonomous for long periods of time without the need to charge the battery.

### 4.5. Battery Discharge

**Figure 22:**
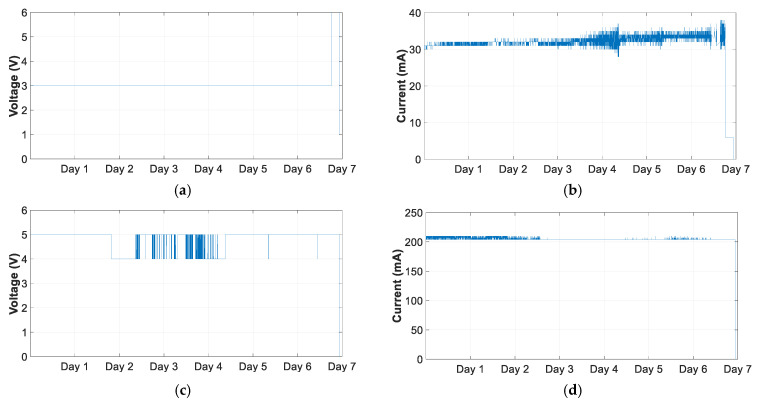
Discharge measurement: (**a**) battery voltage; (**b**) battery current; (**c**) LiPo out voltage; and (**d**) LiPo out current.

The full discharge test has been performed on a fully charged battery. Figure 22 shows the results for the voltages and currents of the battery and at the output of the LiPo controller board. The time required for full discharge was 166.5 h, which ensures a week of operation with only one battery charge without using an SP.

The battery voltage remains constant at 3 V until the point of full discharge. On the other hand, the regulator card maintains an output voltage between 4 and 5 V. The average current consumption is around 33 mA. In this case, it can be seen that the regulator card maintains an output current of between 204 and 210 mA until the end of the charge.

### 4.6. Energy Consumption Comparative

In addition to the wide coverage provided by the LoRaWAN equipment, there is the advantage of the reduced power consumption of these devices. At this point, the consumption of the developed equipment was tested in relation to other wireless technologies.

The comparison was conducted during the month of January 2020 with data taken from THMDL and a Wi-Fi device. The Wi-Fi device tested was an Arduino Wemos D1 mini [84] without a connection to any of the electrical sensors, temperature and humidity sensors or the drive relay. The use of these components would increase the power consumption of the device. It should be noted that the Wemos D1 mini board is one of the boards with the lowest power consumption among those with Internet access via Wi-Fi and the ESP8266 chip. Other boards with this chip have higher power consumption, such as NodeMCU [85], Wemos D1 mini pro [86], Wemos D1 R1 [87], etc.

**Figure 23:**
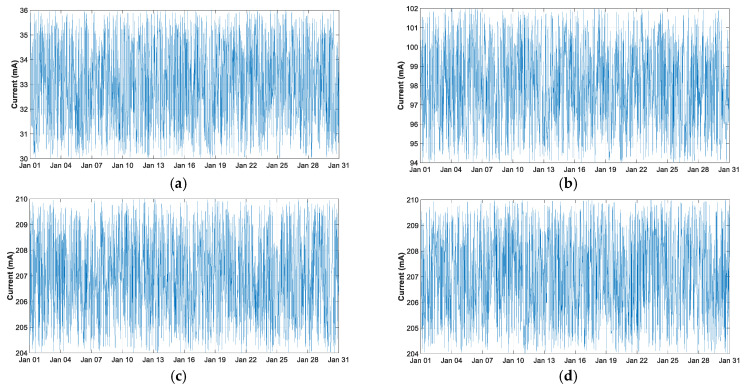
Consumption comparative in January 2020: (**a**) battery current with THMDL connected; (**b**) battery current with Arduino Wemos D1 mini connected; (**c**) LiPo current out with THMDL connected; and (**d**) LiPo current out with Arduino Wemos D1 mini connected.

Figure 23 shows the result of the voltage and current measurements on the battery at the output of the LiPo board. It can be seen that the power consumption of the Wemos D1 mini is approximately three times higher than that of the THMDL, which would be increased by adding the sensors. In addition to this high consumption, the necessary Wi-Fi repeaters or routers would have to be added, as the Wi-Fi coverage is much lower than that offered by the LoRaWAN network, which would increase the final consumption of the whole.

The average THMDL consumption is 33.02 mA, with a standard deviation of 1.76. Wemos D1 mini has a mean of 98.01 mA and a standard deviation of 2.28. The energy consumed by THMDL in January 2020 is 73.6329 Wh and 218.61 Wh for Wemos D1 mini. The total energy consumed in 2020 by THMDL was 869.0219 Wh, corresponding to 2.3762 Wh/day.

As for the output of the LiPo card, it can be seen that, as mentioned above, it maintains an output of between 204 and 210 mA, regardless of the battery consumption. In view of the consumption results, together with the wireless coverage, this supports the use of LoRaWAN technology in systems such as the one developed in this research.

Statistics for the annual consumption of THMDL have been done. Since the average consumption is 33.02 mA and almost constant, the mean is almost equal in all months at around 2.3750, and a standard deviation of 0.0026 is almost zero. The skewness is practically close to zero, with some positive and negative values but in the region of zero, indicating a symmetrical distribution curve. On the other hand, the kurtosis reflects a mesokurtic distribution with a curve with uniformly distributed values in the symmetrical distribution. Table 12 shows the results obtained.

### 4.7. Solar Energy Generated

**Figure 24:**
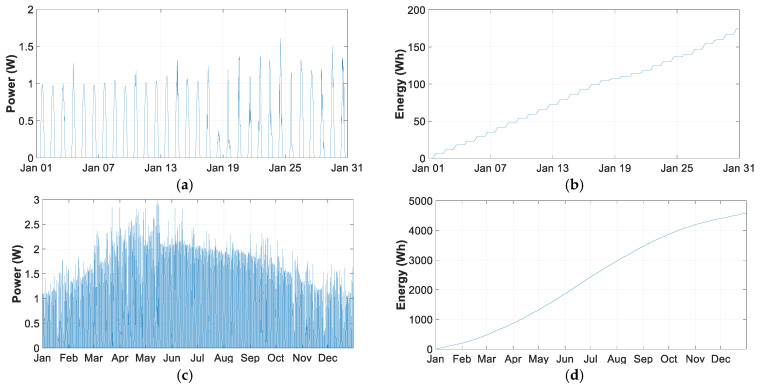
Consumption comparative: (**a**) power obtained in January; (**b**) energy obtained in January; (**c**) power obtained in the year 2020; and (**d**) energy obtained in the year 2020.

This section shows the power and energy generated by the 3 W SP used in THMDL and ECDL. The data were collected during 2020 from one of the THMDLs with SP. Figure 24 shows the energy and power for the month of January 2020, which is the lowest generation month together with December and for the whole year.

The energy generated in January is 202.19 Wh, with a daily average of 6.5266 Wh. The annual photovoltaic generation amounts to 4594.73 Wh and a daily average of 12.5639 Wh.

Table 13 summarises the statistical results of the annual empirical distributions for SP power generation. To compare the results, the monthly and annual average was taken. Thus, the month of maximum generation is June with a mean pf 18.9210 Wh/day. On the other hand, December has the lowest generation with a mean of 6.2873 Wh/day. The generation in December is 33.22% over June and 50.04% over the annual average.

Positive skewness values indicate that the tail of the distribution is longer on the right for values above the mean, and the values are concentrated more to the left of the mean, with only January, November and December located to the right of the mean.

The months of January, November and December present a leptokurtic kurtosis because their coefficient is greater than 3, indicating that the values are concentrated around the mean. The rest of the months are mesokurtic because they have a coefficient lower than 3 and their values are further away from the mean.

### 4.8. Analysis of Consumption, Photovoltaic Generation and Battery Life

Using the data in Table 12 and Table 13, a comparison can be made between the energy consumed by THMDL and SP. Starting from the month of lowest generation, which is December with 194.77 Wh and 6.2873 Wh/day and comparing them with the THMDL consumption of 73.7580 Wh and 2.3808 Wh/day, it can be concluded that the energy generated in the most unfavourable month covers the total daily consumption required. The ratio of generation to consumption is 2.64 times higher.

Considering the results obtained for the total discharge of the battery with an average of 33 mA, 166.5 h were needed, which is much longer than the time needed to recover the sunlight the next day and generate the energy consumed during the hours of no photovoltaic generation.

In this sense, fully discharging the battery would take 6.9375 days, corresponding to 1 day 14.41% of the total charge of the battery in the absence of sunlight. As in the most unfavourable month, there are 6.2873 Wh/day of generation and 2.3808 Wh/day are consumed, the PV generation largely covers the maximum of 14.41% of the battery charge consumed. In this way, the lifetime of the battery is extended to a large extent, as complete charge and discharge cycles are not necessary.

### 4.9. LoRaWAN Measurements

The LoRaWAN was implemented with the network optimisation algorithm developed by Cano-Ortega et al. [8]. The algorithm allows adapting the network parameters in real time in order to obtain the smallest possible ratio of lost packets so as to minimise the loss of information. The algorithm was implemented in the Raspberry that supports the LoRa concentrator.

**Figure 25:**
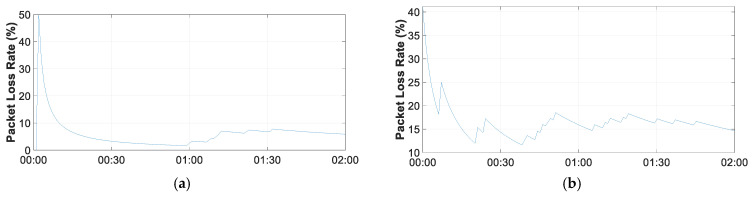
LoRaWAN PLR: (**a**) THMDL measurement and (**b**) ECDL measurement.

Figure 25 shows a part of the measurements made on the network. Two hours of measurements are shown. In the graphs, one can see the changes made by the algorithm marked by the points in the graph, where one can see the change of parameters and the reduction in the rate of loss of information. It should be noted that the location of the devices with respect to the concentrator has a decisive influence on the rate of packets lost. The THMDL device is closer to the concentrator than the ECDL device shown. As can be seen, this has a clear influence on the rate of lost data.

### 4.10. ThingSpeak Integration

As discussed in Section 3.1, data are sent to TTN, and from there, they can be derived to multiple services operating in the cloud. A large number of possible integrations supported by TTN are available. Among them, the integration with the MathWorks ThingSpeak service was chosen to be shown in this research. ThingSpeak allows sending information in its free version of up to four channels of eight fields with a data latency of 15 s. If the needs of the system are greater, it is possible to switch to the paid version, which allows latency times to be reduced to 1 s.

**Figure 26:**
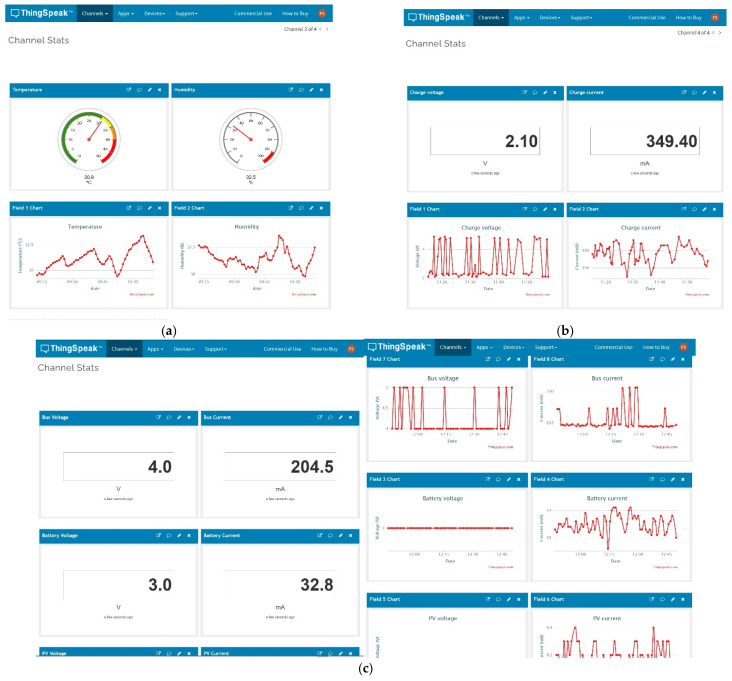
ThingSpeak Integration: (**a**) temperature and humidity data; (**b**) battery USB charge; and (**c**) THMDL battery power supply operation.

The configuration chosen for the LoRaWAN has the following parameters: BW125, SF7, CR4/5, message header 13 bytes and 16 bytes payload. As can be seen in Table 11, the minimum information sending time is 6.7 s, calculated from the European Telecommunications Standards Institute (ETSI) standard [88], complying with the 1% maximum duty cycle rule. As a data sending time of 60 s was chosen, it complies with the current regulations. Figure 26 shows three examples of integration: (a) temperature and humidity data collection; (b) electrical variables of battery charging in USB mode; and (c) data collection of the electrical variables of the THMDL in the battery-only version.

### 4.11. Future Work

The high power of LoRaWAN is limited by the 1% duty cycle time for sending data, which for the payload used in this research is 6.7 s, together with the limitation of sending data to ThingSpeak every 15 s in the free version may limit the message sending. If the payload is increased with more sensors added to THMDL or ECDL, the minimum time for sending data would increase. In this sense, a future line of research would be to develop a LoRa concentrator that works outside the LoRaWAN specification using technology that avoids data delivery limitations for systems with data latency of less than 1% of the duty cycle. Complementing the previous line, higher data latency systems should be used in the cloud, such as Google’s Firebase, which allows up to 0.2 s of data upload time.

It would also be interesting to create a web page and an application for mobile devices where the monitored and controlled installations are collected in real time. Finally, further studies should provide algorithms with machine learning intelligence to improve its performance through the experiences collected during the operation of the installations.

## 5. Conclusions

This research develops a complete irrigation system based on wireless communication over a LoRaWAN. It meets the objectives of low power consumption, small size, integration, modular design, fault response, operational safety and low price. These objectives have been achieved by overcoming a number of technical challenges, including component selection, modular design, evaluation of alternatives and PCB design to integrate the components used in the THMDL and ECDL.

LoRaWAN has a wide coverage of up to 10 km, with coverage in urban environments reaching up to 5 km. In addition, up to 1000 devices can be integrated with a single gateway or hub, reducing the infrastructure to be installed. If Wi-Fi, Bluetooth, etc., devices were used, a multitude of repeater devices would be required, which would greatly complicate the complexity of the installation. On the other hand, the power consumption of LoRaWAN devices is extremely low compared to Wi-Fi and similar devices. This reduced power consumption increases battery life and extends system uptime.

The system incorporates battery management for low battery warnings with an adjustable warning level. The irrigation routine allows the minimum moisture level for watering to be set. This routine also incorporates a rainfall forecast query that offers the possibility of not watering if the probability of rainfall is higher than the set value and no watering message is sent. The system is equipped with redundant message sending, which minimises the loss of information in the system. On the other hand, all minimum battery voltage levels, minimum rain probability, minimum humidity and waiting time for receiving and sending messages can also be set. This makes for a dynamic, robust and fault-tolerant system that can be installed in a multitude of locations.

A comparison of the prototypes used with other wireless technologies was performed. In this sense, the average consumption of THDML is 2.3762 Wh/day, and the average consumption of the Wi-Fi device studied is 7.0519 Wh/day without sensors and other components, which is 2.96 times higher. The generation of the SP used is 6.2873 Wh/day in the month of lowest generation, which is well within the THMDL average consumption of 2.3808 Wh/day, which is 14.41% of the battery capacity. This contributes to decreasing the charge and discharge cycles of the battery and extending the battery life.

The use of TTN opens up a wide range of possibilities for the development of system functionalities and adaptation to the needs of each implementation. TTN integrates a large set of cloud services, such as the one presented at the end of the Results section (ThingSpeak). In each implementation of the system, the needs of each problem can be studied, and the most suitable service can be used to offer the best solution. On the other hand, the system can also be reprogrammed by adding new functionalities to improve its performance characteristics. By using Arduino as the basis for the devices, the system benefits from the advantages of the open-source platform of this family.

## Figures and Tables

**Table 1:** Comparison of the Arduino family.

Component	Surface (mm^2^)	Microcontroller	Current Consumption (mA)	Flash Memory (kB)	Clock Speed (MHz)	Unit Price (€)
Arduino Uno [53]	3663.24	ATmega328P	46	32	16	20.00
Arduino Mega [54]	5421.17	ATmega2560	93	256	16	35.00
Arduino Nano [52]	810.00	ATmega328	15	32	16	20.00
Arduino Micro [55]	864.00	ATmega32U4	15	32	16	18.00

**Table 2:** Comparison of LoRa end-devices.

Component	Surface (mm^2^)	Current Consumption (A)	RSSI Range (dBm)	Sensitivity (dBm)	Blocking Immunity	Unit Price (€)
Lopy4 [65]	1100.00	Rx 12 mA–0.2 µA register retention	−126	−148	High	33.06
Monteino [63]	240.05	RX 10.3 mA–200 nA register retention	−127	−148	Excellent	22.95
Libelium [63]	775.00	RX 10.3 mA–200 nA register retention	−127	−148	Excellent	32.35
MKR WAN 1310 [62]	1693.75	Rx 23.5 mA	−117.5	−133.5	High	33.00
Dragino LoRa Bee [66]	775.00	RX 10.3 mA–200 nA register retention	−127	−148	Excellent	14.50

**Table 3:** Comparison of LoRa gateways and concentrators.

Component	Number of Channels	Communication Paths	Number of LoRa Devices	Unit Price (€)
Dragino OLG01 [68]	1	Ethernet—Wi-Fi—3G/4G	300	85.79
Dragino OLG02 [69]	2	Ethernet—Wi-Fi—3G/4G	300	95.89
LoRa concentrator [67]	10	Ethernet—Wi-Fi provided by Raspberry	1000	100.19
LoRa GPS Hat [70]	1	Ethernet—Wi-Fi provided by Raspberry	300	35.90

**Table 4:** Comparison of electrical sensors.

Component	Measured Variable	Surface (mm^2^)	Price (€)
FZ0430 [71]	Voltage	378	1.73
ACS712 [72]	Current	420	1.28
IN219 [73]	Voltage, current, PF and power	2211	1.70

**Table 5:** Comparison of temperature and humidity sensors.

Sensor	Humidity Accuracy (%)	Temperature Accuracy (°C)	Supply Voltage (V)	Energy Consumption (µW)	Humidity Range (%)	Temperature Range (°C)	Interface
SHT10 [74]	±4.5	±0.5	2.4–5.5	80	0–100	−40/125	SBus
SHT11 [74]	±3	±0.4					
SHT15 [74]	±2	±0.3					
SHT20 [75]	±2	±0.3	2.1–3.6	3.2	0–100	−40/125	I2C PWM SDM
SHT21 [75]	±2	±0.3					
SHT25 [75]	±1.8	±0.2					
SHT30 [76]	±2	±0.2	2.15–5.5	4.8	0–100	−40/125	I2C
SHT31 [76]	±2	±0.2					
SHT35 [76]	±1.5	±0.1					
DHT11 [77]	±5	±5	3.3–5	100	20–80	0–50	Digital pin
DHT22 [78]	±5	±5	3.3–5	100	0–100	−40/125	Digital pin

**Table 6:** Comparison of charge regulators.

Sensor	V_in_ Solar (V)	I_charge_ (mA)	I_load_ (mA)	V_batt_ (V)	V_source_ (%)	V_destination_ (°C)
Lipo Rider Pro [79]	5	500	1000	4.2	5	5
Lipo Rider Plus [80]	5	250	250	100	3.3	3.3
Lipo Rider v1.3 [81]	5	800	600	4.2	5	5

**Table 7:** Cost of components for the THMDL with a battery power supply.

Description	Number	Unit Price (€)	Total (€)
Microcontroller Arduino Nano	2	20.00	40.00
Dragino LoRa Bee	1	14.50	14.50
INA219	1	1.70	1.70
Battery	1	32.96	32.96
PCB board	1	0.40	0.40
SHT30 sensor	1	6.80	6.80
Box container	1	2.54	2.54
Auxiliary material and wiring	-	1.05	1.05
		Total cost	99.95

**Table 8:** Cost of components for the THMDL with a battery and solar panel power supply.

Description	Number	Unit Price (€)	Total (€)
Microcontroller Arduino Nano	2	20.00	40.00
Dragino LoRa Bee	1	14.50	14.50
INA219	3	1.70	5.10
Battery	1	32.96	32.96
Solar panel	1	12.28	12.28
Li-Po Rider Pro	1	15.33	15.33
PCB board	1	0.40	0.40
SHT30 sensor	1	6.80	6.80
Box container	1	3.02	3.02
Auxiliary material and wiring	-	1.27	1.27
		Total cost	131.66

**Table 9:** Cost of components for the ECDL with a battery power supply.

Description	Number	Unit Price (€)	Total (€)
Microcontroller Arduino Nano	2	20.00	40.00
Dragino LoRa Bee	1	14.50	14.50
INA219	1	1.70	1.70
Battery	1	32.96	32.96
PCB board	1	0.40	0.40
Relay	1	0.27	0.27
Box container	1	2.54	2.54
Auxiliary material and wiring	-	1.05	1.05
		Total cost	93.62

**Table 10:** Cost of components for the ECDL with a battery and solar panel power supply.

Description	Number	Unit Price (€)	Total (€)
Microcontroller Arduino Nano	2	20.00	40.00
Dragino LoRa Bee	1	14.50	14.50
INA219	3	1.70	5.10
Battery	1	11.50	11.50
Solar panel	1	12.28	12.28
Li-Po Rider Pro	1	15.33	15.33
PCB board	1	0.55	0.55
Relay	1	0.27	0.27
Box container	1	3.02	3.02
Auxiliary material and wiring	-	1.27	1.27
		Total cost	122.28

**Table 11:** Airtime parameters for the LoRaWAN in EU868 zone.

Data Rate	Parameters	Airtime	Duty Cycle (1% max)		Fair Access Policy		
			Time (s)	Msg/Hour	Avg/s	Avg/Hour	Msg/24h
DR5	SF7-BW125	66.8	6.7	538	192.4	18.7	448
DR4	SF8-BW125	123.4	12.3	291	355.4	10.1	243
DR3	SF9-BW125	226.3	22.6	159	651.8	5.5	132
DR2	SF10-BW125	441.6	41.2	87	1185.5	3.0	72
DR1	SF11-BW125	905.2	90.5	39	2607.0	1.4	33
DR0	SF12-BW125	1646.2	164.7	21	4742.2	0.8	18
DR6	SF6-BW250	33.4	3.3	1077	96.2	37.4	897

**Table 12:** Descriptive statistics of the power THMDL consumption in year 2020.

Month	Energy Generated (Wh)	Sample Daily Mean (Wh)	Sample Variance (Wh^2^)	Sample Skewness (Wh^3^)	Sample Kurtosis (Wh^4^)
January	73.6329	2.3768	0.0026	0.0143	1.7544
February	68.7263	2.3715	0.0025	0.0418	1.8301
March	73.5223	2.3764	0.0025	−0.0118	1.8161
April	71.2977	2.3782	0.0026	−0.0391	1.7955
May	73.6111	2.3761	0.0025	0.0044	1.8280
June	71.2468	2.3765	0.0025	0.0084	1.8135
July	73.4652	2.3714	0.0025	0.0613	1.8834
August	73.5958	2.3756	0.0026	0.0163	1.7741
September	71.3757	2.3808	0.0025	−0.0511	1.8063
October	73.5985	2.3757	0.0025	−0.0037	1.8309
November	71.1906	2.3746	0.0025	0.0261	1.8079
December	73.7580	2.3808	0.0025	−0.0592	1.8529
Year	869.0216	2.3762	0.0025	0.0008	1.8140

**Table 13:** Descriptive statistics of the power SP generation in year 2020.

Month	Energy Generated (Wh)	Sample Daily Mean (Wh)	Sample Variance (Wh^2^)	Sample Skewness (Wh^3^)	Sample Kurtosis (Wh^4^)
January	202.19	6.5266	0.2142	1.3553	3.4122
February	276.04	9.5255	0.2751	0.9939	2.3714
March	394.35	12.7469	0.3600	1.0546	2.6887
April	452.60	15.0974	0.4086	1.0099	2.5663
May	538.46	17.3816	0.4194	0.7060	1.9756
June	567.23	18.9210	0.4213	0.4987	1.6505
July	542.87	17.5239	0.3927	0.4846	1.5712
August	496.76	16.0353	0.3786	0.5693	1.6509
September	399.08	13.3120	0.3475	0.8171	2.0916
October	326.09	10.5262	0.2974	0.9640	2.3564
November	204.23	6.8126	0.2220	1.3580	3.4074
December	194.77	6.2873	0.2103	1.3909	3.5401
Year	4594.73	12.5639	0.3502	1.0595	2.7639

## Abbreviations

The following abbreviations are used in this manuscript.

AN	Arduino Nano
BW	Bandwidth
CR	Code rate
DLB	Dragino LoRa Bee
ECDL	Electrovalve Control Device for LoRaWAN
GPRS	General Packet Radio Service
I2C	Inter-Integrated Circuit
IFTTT	If This, Then That
IoT	Internet of Things
LiPo	Lipo Rider Pro
LoRa	Long range
LoRaWAN	Long-range wide-area network
LPWAN	Low-power wide-area network
MQTT	Message Queue Telemetry Transport
NB-IoT	Narrow-band Internet of Things
PCB	Printed Circuit Board
SF	Spread factor
SP	Solar panel
THMDL	Temperature and Humidity Measurement Device for LoRaWAN
TTN	The Things Network
Wi-Fi	Wireless Fidelity
